# The Clinical Characteristics of Other HLA-B Types in Chinese Ankylosing Spondylitis Patients

**DOI:** 10.3389/fmed.2020.568790

**Published:** 2021-01-08

**Authors:** Xinyu Wu, Jialing Wu, Xiaomin Li, Qiujing Wei, Qing Lv, Pingping Zhang, Xuqi Zheng, Zena Chen, Shuangyan Cao, Liudan Tu, Jieruo Gu

**Affiliations:** ^1^Department of Rheumatology, The Third Affiliated Hospital of Sun Yat-Sen University, Guangzhou, China; ^2^Department of Pediatrics, The Third Affiliated Hospital of Sun Yat-Sen University, Guangzhou, China

**Keywords:** ankylosing spondylitis, HLA-B40, HLA-B46, HLA-B genotype, peripheral joint involvement

## Abstract

HLA-B27 has an established relationship with the development of ankylosing spondylitis (AS). After reviewing the HLA-B genotype from 407 Chinese subjects (318 patients and 89 sex-matched controls), we found that 252 patients and 32 controls were HLA-B27(+) and that HLA-B^*^27:04 was the dominant HLA-B27 subtype (*N* = 224). In all participants, HLA^*^27:04 homozygous were only detected in two patients. In the HLA-B27(+) group, HLA-B40 was observed in 51 cases and one control (*p* < 0.05, OR = 7.87, 95% CI 1.05–59.0); of these, the most genotype was HLA-B^*^27:04/B^*^40:01(*N* = 38). Two hundred thirty-nine patients' clinical information was recorded. Cases with HLA-B27/B46 had more peripheral joint involvement (OR = 3.95, 95% CI 1.77–8.79) in HLA-B27(+) AS. HLA-B^*^15:02 may be a significant risk element to peripheral joint involvement (*p* < 0.05) in HLA-B27(−) patients. Therefore, we believe HLA-B^*^40:01, HLA-B^*^46:01, and HLA-B^*^15:02 can be the test indicators for AS diagnostic value.

## Introduction

Human leukocyte antigen (HLA)-B27 is the most critical gene in ankylosing spondylitis (AS). About 90–95% of AS cases were HLA-B27 positive, while only 1–2% of HLA-B27 positive persons can develop to AS (1, 2). Results showed that the occurrence of AS with HLA-B27 appeared in family aggregation. Among the first-degree relatives of HLA-B27 positive AS, the prevalence is 10–30% (3). Above 45 HLA-B27 subtypes, like B^*^27:02, B^*^27:10, and B^*^27:15, were found to be associated with AS, and their distribution varied in different populations (4, 5). B^*^27:04 is the primary subtype in the Chinese Han population (6), whereas the Caucasian people are dominated by the B^*^27:05 (4). On the contrary, B^*^27:06 and B^*^27:09 are unrelated to AS. Previous research found homozygous B^*^27:04 can affect AS susceptibility but not its clinical manifestations and functional disability (7, 8). How about HLA-B27 heterozygote with other HLA-B alleles in AS? Our studies aimed to evaluate the influence of heterozygous HLA-B27 on the clinical manifestations of AS patients.

## Methods

### Study Subjects

Three hundred eighteen Chinese Han patients and 89 sex-matched controls were recruited from the hospitals in Guangdong Province of China. All patients were older than 18 years old and met the 1984 modified New York criteria for AS (9). Two hundred thirty-nine patients had their clinical information collected by two trained rheumatologists during a face-to-face interview at the study visit. Clinical information included peripheral manifestations (uveitis, peripheral joint involvement, dactylitis, and enthesitis), onset age, body mass index (BMI), Bath Ankylosing Spondylitis Disease Activity Index (BASDAI), and Bath Ankylosing Spondylitis Functional Index (BASFI). We also collected past and current medications, including non-steroidal anti-inflammatory drugs (NSAIDs), corticosteroids, disease-modifying antirheumatic drugs (DMARDs), and biologic agents. The general information included age, gender, and smoking (current) and drinking history (current). According to the 2009 ASAS classification criteria (either axial or peripheral) (10), patients without any peripheral manifestations (uveitis, peripheral joint involvement, dactylitis, and enthesitis) were classified as the axial AS (axAS). Controls were free of any history of rheumatic disease. Written informed consent was obtained from all the subjects. The ethics committee of the Third Affiliated Hospital of Sun Yat-Sen University approved our study. All participants gave written informed consent before enrollment.

### HLA-B Genotype

Genomic DNA was extracted from peripheral blood using a standard salting-out method. All of the individuals were genotyped for HLA-B loci using the polymerase chain reaction sequence-based typing (PCRS-BT) method. Briefly, we performed locus-specific PCR amplification and bidirectional Sanger sequencing of HLA-B exons 2, 3, and 4. Amplification and sequencing of relevant exons was performed using “in-house” primers. Sequencing was performed on a 3730XL DNA analyzer (Applied Biosystems, Foster City, CA, USA). The typing results were accomplished using uTYPE v6.0 software (One Lambda, Canoga Park, CA, USA) against the IMGT/HLA database. When encountering ambiguous genotyping results (several genotypic combinations perform identically on sequencing results), alleles were assigned by referring to the most common alleles in the Chinese population (11).

### Statistical Analysis

**Figure 1:**
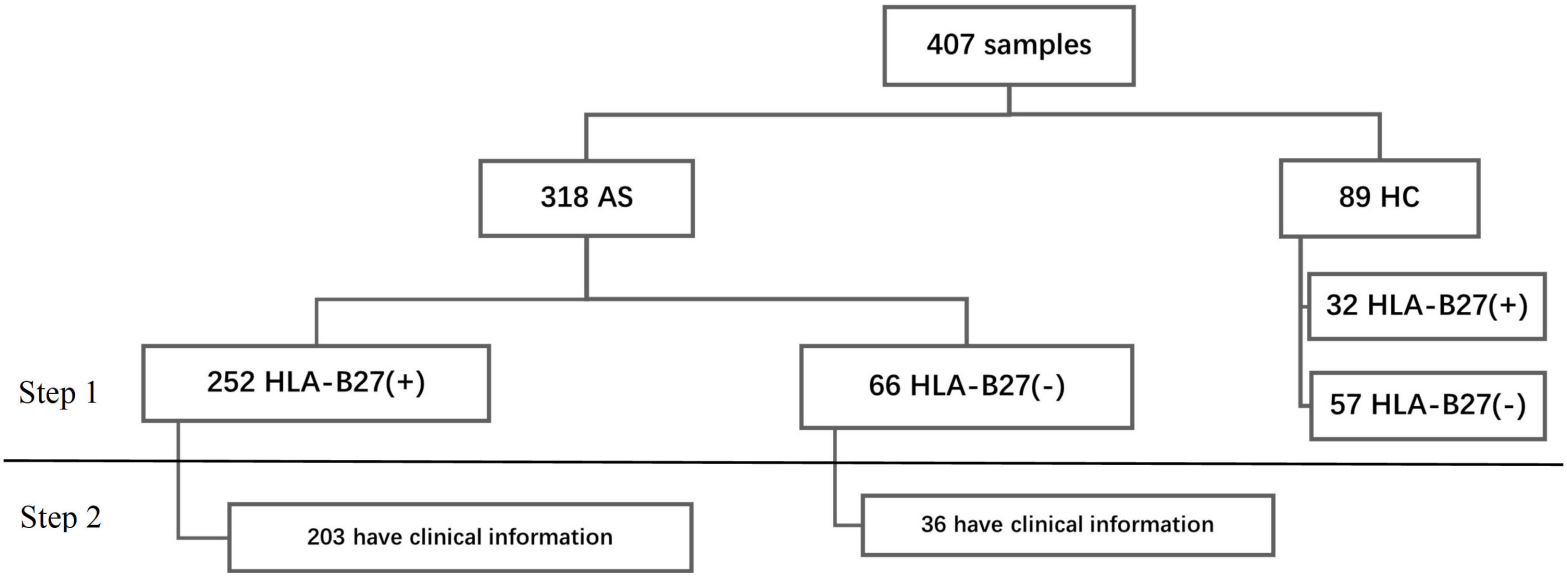
Study flow chart.

We analyzed the data in two steps. In step 1, we analyzed the HLA-B types in all participants. Then we researched the relationship with HLA-B types and clinical phenotype (Figure 1). For continuous variables, we calculated mean ± standard deviation (SD) and percentage for categorical variables. We performed Student's *t*-test or rank-sum test to make group comparisons for continuous data and chi-squared tests for categorical variables (Fisher's exact test where appropriate). All contrasts were bilateral and considered significant when *p* < 0.05. Data were collected, processed, and analyzed using the Statistical Package for the Social Sciences (SPSS) software v.19. The heatmaps were drawn by R software v3.6.1.

## Results

### Step 1

#### The HLA-B Genotypes Distribution in all Samples

**Figure 2:**
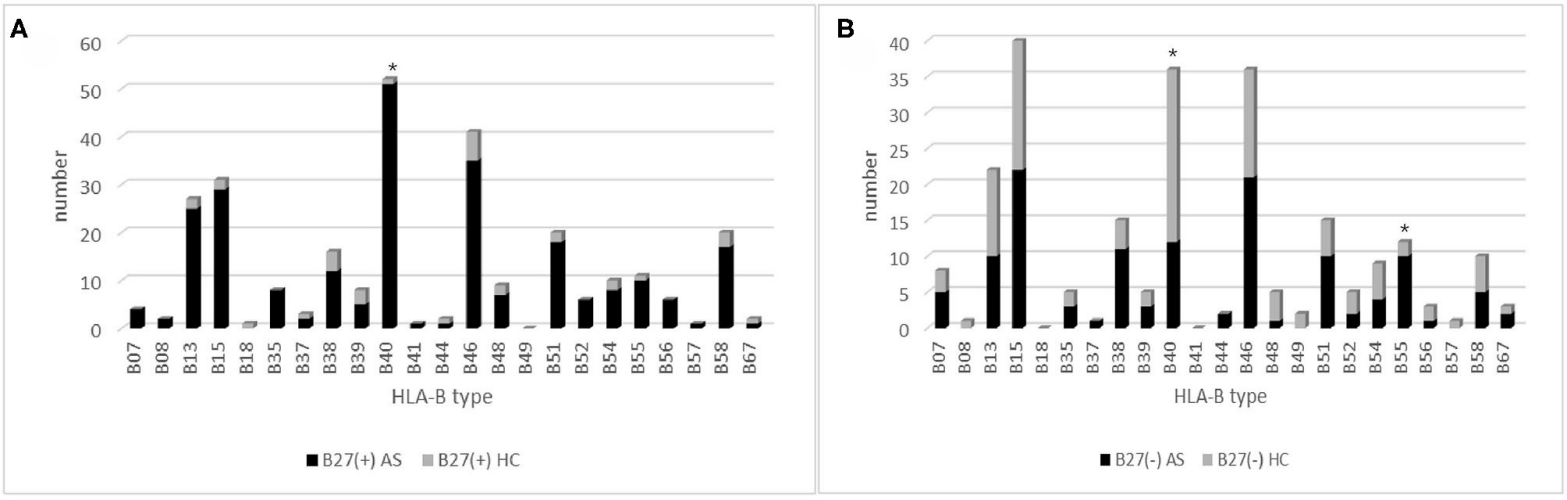
The HLA-B type distribution in HLA-B27(+) and HLA-B27(−) group: **(A)** HLA-B type distribution in HLA-B27(+) patients and HLA-B27(+) controls, and **(B)** HLA-B type distribution in HLA-B27(−) patients and HLA-B^(−)^ controls. Chi-squared test were used to determine significance (*p < 0.05). AS, ankylosing spondylitis; HC, health control.

A total of 407 subjects were analyzed using HLA-B typing including 318 AS and 89 sex-matched controls (Figure 1) from January 2016 to September 2020. As shown in Table 1, the mean age of AS patients was 29.55 ± 8.83 years old and controls was 39.86 ± 18.03 years old. The AS patients were younger than controls (*p* < 0.05). The main age group of patients was under 40 years old. After HLA-B typing, we found 24 low-resolution HLA-B types and 55 high-resolution HLA-B subtypes in all participants, including eight homozygous and 399 heterozygous. The major HLA-B type was HLA-B27. Two hundred fifty-two (79.25%) cases and 32 (35.96%) controls (*p* < 0.05) were HLA-B27(+). Other HLA-B-type distributions are shown in Figure 2.

**Table 1:** The basic information of all AS and controls (step 1).

	**AS (*N =* 318)**	**HC (*N =* 89)**	***p***	**OR (95% CI)**
Sex			0.483^a^	
Male, *n* (%)	220 (69.2)	65 (73.0)		
Female, *n* (%)	98 (30.8)	24 (27.0)		
Age (years), mean ± SD	29.55 ± 8.83	39.86 ± 18.03	<0.001^b^	
<20	33	5		
20–40	224	41		
40–60	31	18		
60–80	1	9		
B27(+), *n* (%)	252 (79.2)	32 (35.96)	<0.001^a^	6.801 (4.081–11.335)

*AS, ankylosing spondylitis; HC, health controls; B27 HLA-B27; OR, odds ratio; CI, confidence interval*;

a *Chi-squared test*;

b *Student's t-test*.

**Figure 3:**
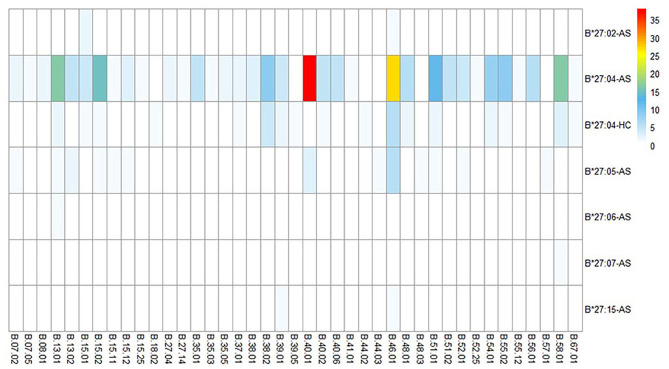
HLA-B alleles heatmap of different HLA-B27 subtypes combined with other HLA-B subtypes. Each square represents a genotype, like HLA-B*27:04/B*40:01. AS, ankylosing spondylitis; HC, health control.

In 252 B27(+) patients and 32 B27(+) controls, HLA-B^*^27:04 was found in 224 cases (88.89%) and in all 32 controls (100%), respectively (*p* > 0.05). HLA-B^*^27:05 was detected in 21 cases (8.3%) but not found in controls. We also observed another HLA-B subtype (one HLA-B^*^27:07, one HLA-B^*^27:06, two HLA-B^*^27:15, three HLA-B^*^27:02, and one HLA-B^*^27:14) in patients (Table 2). The one with HLA-B^*^27:14 was a HLA-B^*^27:04 heterozygote (HLA-B^*^27:04/B^*^27:14). Fifty-one cases (15.87%) and one control (3.13%) carried B27/B40 (*p* = 0.018); the majority genotype was HLA-B^*^27:04/B^*^40:01 (*N* = 38, Figure 3). In HLA-B^*^27:04 carriers, the HLA-B^*^40:01 was also associated with AS (*p* = 0.024). In 21 HLA-B^*^27:05 patients, HLA-B^*^27:05/B^*^46:01 was the most HLA-B genotype (*N* = 6, 28.6%) (Figure 3). Between HLA-B^*^27:04 and HLA-B^*^27:05 patients, the distribution of the HLA-B^*^40:01 and HLA-B^*^46:01 had no significant difference. Another HLA-B27 subtype genotype is shown in Figure 3.

**Table 2:** The HLA-B27 subtypes in all B27(+) groups and the associated HLA-B types in B27(+) and B27(−) groups (step 1).

	**B27(+)**				**B27(−)**			
	**AS**	**HC**	***p***	**OR (95% CI)**	**AS**	**HC**	***p***	**OR (95% CI)**
*N*	252	32			66	57		
Sex			0.166				0.171	
Male, *n* (%)	184 (73.0)	27 (84.4)			36 (54.55)	38 (66.67)		
Female, *n* (%)	68 (27.0)	5 (15.6)			30 (45.45)	19 (33.33)		
Age (years), mean ± SD	29.3 ± 8.4	41.2 ± 20.4	0.003		30.7 ± 10.2	39.0 ± 16.4	0.003	
Homozygote	2 (0.8)				4 (6.0)	2 (3.5)		
HLA-B27 subtype, *n* (%)								
B^*^27:04	224 (88.9)	32 (1.00)	0.095					
B^*^27:02,	3 (1.2)							
B^*^27:05	21 (8.3)							
B^*^27:06	1 (0.4)							
B^*^27:07	1 (0.4)							
B^*^27:15	2 (0.8)							
Other HLA-B types, *n* (%)								
B40	51 (20.2)	1 (3.1)	0.018	7.87 (1.05-59.0)	12 (18.18)	24 (42.11)	0.004	0.306 (0.135–0.692)
B^*^40:01	41 (16.3)	0	0.028		9 (13.64)	21 (36.84)	0.003	0.271 (0.112–0.656)
B55	10 (4.0)	1 (3.1)	0.800		10 (15.15)	2 (3.51)	0.03	4.911 (1.029–23.442)
B^*^55:02	9 (3.6)	1 (3.1)	0.900		9 (13.64)	2 (3.51)	0.05	
B15	29 (11.5)	2 (3.1)	0.550		22 (33.33)	18 (31.58)	0.84	
B^*^15:17					0	7 (12.28)	0.011	

**Figure 4:**
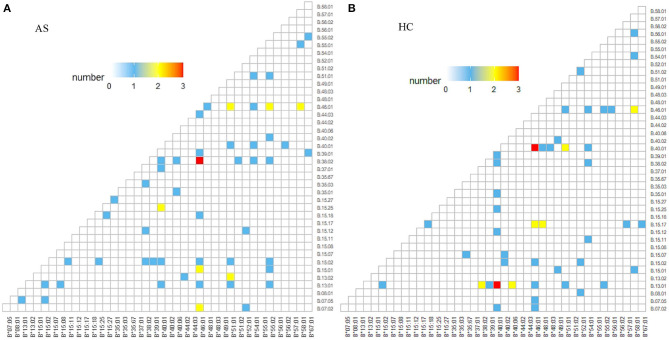
HLA-B alleles heatmap of HLA-B27(−) group. Each square represents a genotype, like HLA-B*40:01/B*46:01: **(A)** HLA-B alleles heatmap of HLA-B27(−) AS patients, and **(B)** HLA-B alleles heatmap of HLA-B27(−) controls. AS, ankylosing spondylitis; HC, health control.

Between 66 B27(−) patients and 57 B27(−) controls (Table 2), HLA-B40 was detected in 12 cases (18.18%) and 24 controls (42.11%) (*p* = 0.004). There was also a significant difference in HLA-B55 between the two groups (*p* = 0.03). At the high-resolution level, we found the number of HLA-B^*^40:01 and HLA-B^*^15:17 was significantly higher in the control group as compared to the case group (*p* < 0.05). The number range of every HLA-B heterozygous genotypes was 1 to 3 (Figure 4).

### Step 2

#### Comparisons of the Clinical Characteristics Between B27(+) and B27(−) AS Patients

Two hundred thirty-nine patients had detailed clinical information, including 203 B27(+) and 36 B27(−) patients. As observed in Table 3, a significant difference was found in sex between two groups (*p* = 0.042), with more male participants in B27(+) patients. However, there was no statistical significance in the current age, age at symptom onset, family aggregation, smoking status, alcohol consumption, BMI, BASDAI, BASFI, peripheral manifestations (uveitis, peripheral joint involvement, dactylitis, and enthesitis), and medications. In B27(+) patients, we also compared clinical characteristics between HLA-B*27:04 and HLA-B*27:05 cases. We did not find any significant difference (Table 4).

**Table 3:** Demographic and disease characteristics of the B27(+) and B27(−) AS patients (step 2).

	**B27(+)**		**B27** ^****(−)****^		***p***
	***N* = 203**	**Mean ± SD or %**	***N* = 36**	**Mean ± SD or %**	
Gender, male	147	72.4	20	55.6	0.042^a^
Age (years)	203	29.66 ± 8.57	36	29.6 ± 11.22	0.990^b^
Onset age (years)	203	22.39 ± 7.58	36	24.39 ± 8.97	0.158^b^
Family history	48	24.1	7	21.9	0.395^a^
Current smoking	30	14.9	3	8.3	0.319^a^
Alcohol consumption	12	5.9	1	2.8	0.715^a^
Body mass index (kg/m^2^)	200	21.31 ± 3.01	36	21.37 ± 2.94	0.913^b^
axAS	118	58.1	18	50.0	0.364^a^
Uveitis	34	16.7	5	13.9	0.669^a^
B*27:04	29				
B*27:05	3				
B*27:02	2				
Enthesis	39	19.2	7	19.4	0.974^a^
B*27:04	34				
B*27:05	4				
B*27:02	1				
Peripheral joint involvement	48	23.6	9	25.0	0.860^a^
B*27:04	40				
B*27:05	5				
B*27:15	1				
B*27:02	2				
Dactylitis	9	4.4	1	2.8	0.995^a^
B*27:04	8				
B*27:02	1				
BASDAI	177	2.74 ± 1.86	33	2.82 ± 1.7	0.807^b^
BASFI	178	1.45 ± 2.29	33	1.01 ± 1.74	0.292^b^
NSAIDs use (only)	36	17.7	10	2.8	0.159^a^
DMARDs use					
Methotrexate (ever)	14	6.9	0		0.215^a^
Sulfasalazine (ever)	80	39.4	8	22.2	0.049^a^
Biological therapy (ever)	66	32.5	6	16.7	0.056^a^

*axAS, axial ankylosing spondylitis; BASDAI, Bath Ankylosing Spondylitis Disease Activity Index; BASFI, Bath Ankylosing Spondylitis Functional Index; NSAIDs, Non-Steroidal Anti-inflammatory Drugs; DMARDs, disease-modifying antirheumatic drugs*;

a *Chi-squared test*,

b *Student's t-test*.

**Table 4:** Disease characteristics of B^*^27:04 and B^*^27:05 AS patients (step 2).

	B^*^27:04	B^*^27:05	***p***
Number	179	17	
Age, mean ± SD	29.41 ± 8.24	31.00 ± 10.88	>0.05^b^
Age onset, mean ± SD	22.06 ± 7.14	24.53 ± 8.57	>0.05^b^
Sex			>0.05^a^
Male, *n* (%)	128 (71.5)	15 (88.2)	
Female, *n* (%)	51 (28.5)	2 (11.8)	
Peripheral manifestations			
Peripheral joint involvement, *n* (%)	40 (22.3)	5 (29.4)	>0.05^a^
Uveitis, *n* (%)	29 (16.2)	3 (17.6)	>0.05^a^
Enthesis, *n* (%)	34 (19.0)	4 (23.5)	>0.05^a^
Dactylitis, *n* (%)	8 (4.4)		
BASDAI, mean ± SD	2.34j ± 1.73	1.36 ± 2.16	>0.05^b^
BASFI, mean ± SD	1.56 ± 1.40	0.21 ± 0.31	>0.05^b^

*BASDAI, Bath Ankylosing Spondylitis Disease Activity Index; BASFI, Bath Ankylosing Spondylitis Functional Index*;

a *Chi-squared test*;

b *Student's t-test*.

#### The Low-Resolution HLA-B Genotypes Distribution in Different Peripheral Manifestations

**Figure 5:**
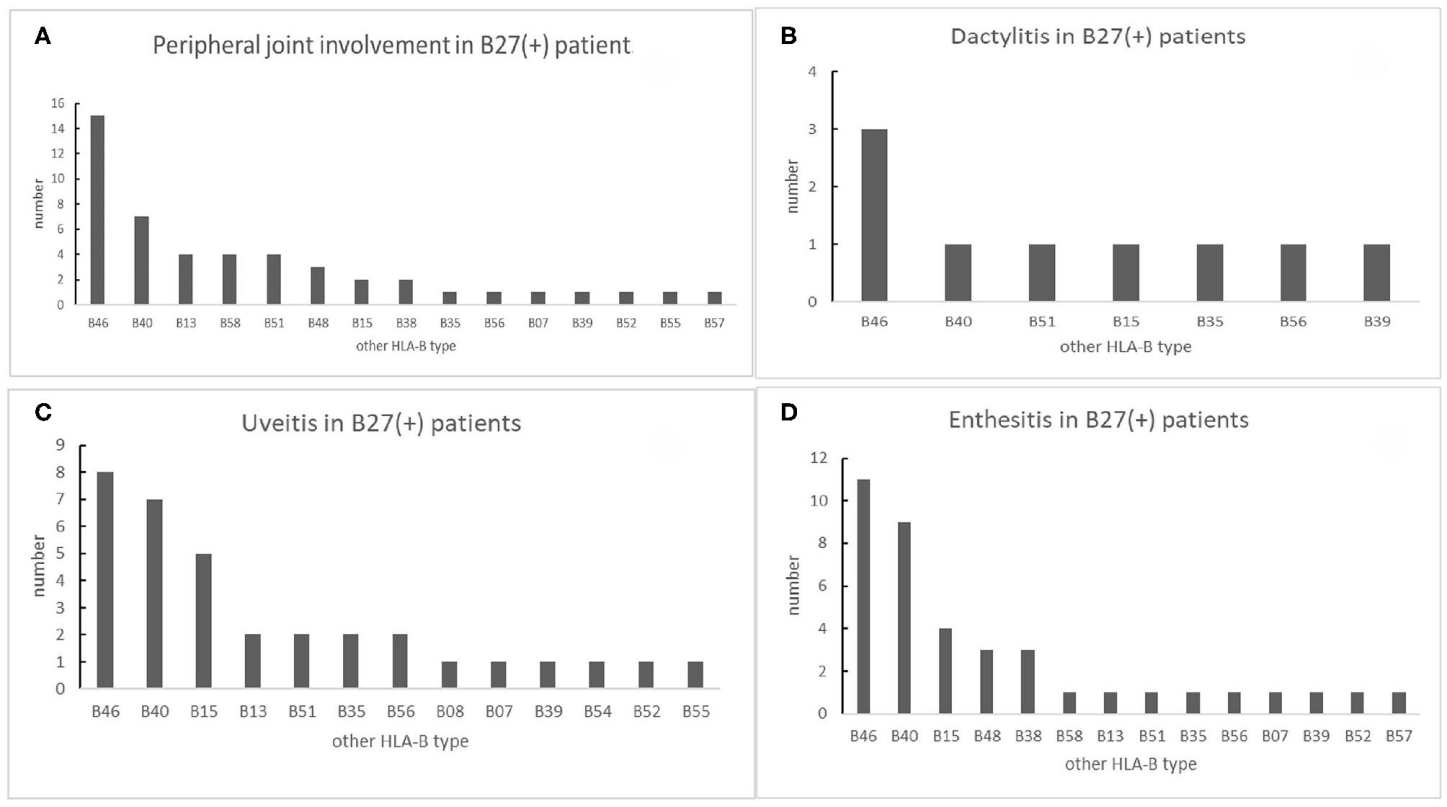
The number of other HLA-B types shown in B27(+) patients with different peripheral manifestations: **(A)** number of other HLA-B types shown in B27(+) patients with peripheral joint involvement, **(B)** number of other HLA-B types shown in B27(+) patients with dactylitis, **(C)** number of other HLA-B types shown in B27(+) patients with uveitis, and **(D)** number of other HLA-B types shown in B27(+) patients with enthesis.

We analyzed the low-resolution HLA-B genotypes of B27(+) patients. In 48 peripheral joint involvement patients with B27(+), 15 cases (31.25%) carried HLA-B46. HLA-B40 was observed in seven cases (14.58%). HLA-B58, HLA-B51, and HLA-B13 were detected in four cases (8%), respectively for each type (Figure 5A). In 39 B27(+) patients with enthesis (Figure 5D), HLA-B46, HLA-B40, and HLA-B15 were found in 11 cases (28.21%), nine cases (23.08%), and four cases (10.26%), respectively. As shown in Figure 5C, in 34 B27(+) patients with uveitis, eight cases (23.53%) carried HLA-B46. HLA-B40 and HLA-B15 were detected in seven cases (20.59%) and five cases (14.71%), respectively. There were nine B27(+) patients with dactylitis (Figure 5B), and HLA-B46 was observed in three cases (33.33%). Compared with HLA-B40 and other HLA-B types in B27(+) patients, HLA-B46 had relationships with peripheral joint involvement and enthesis, respectively (Table 5).

**Table 5:** Association of HLA-B40 and HLA-B46 with peripheral joint involvement in B27(+) AS patients (step 2).

	**Peripheral joint involvement**	**Without peripheral joint involvement**	***p***	**OR (95% CI)**	**Enthesitis**	**Without enthesitis**	***p***	**OR (95% CI)**
B27(+) AS patients, *n*	48	155	0.002		39	164	0.035	
B27/B40	7	39			9	37		
B27/B46	15	16		3.95 (1.77–8.79)	11	20		2.83 (1.22–6.55)
Other HLA-B types	26	100			19	107		
B27:04 AS patients, *n* (%)			0.007^e^				0.037^e^	
B^*^27:04/B^*^40:01 (*N =* 35)	6	29			6	29		
B^*^27:04/B^*^46:01 (*N =* 24)	12	12		4.83 (1.47–15.87)	10	14		3.45 (1.04–11.42)

e Chi-squared test between B^*^27:04/B^*^40:01 and B^*^27:04/B^*^46:01.

**Figure 6:**
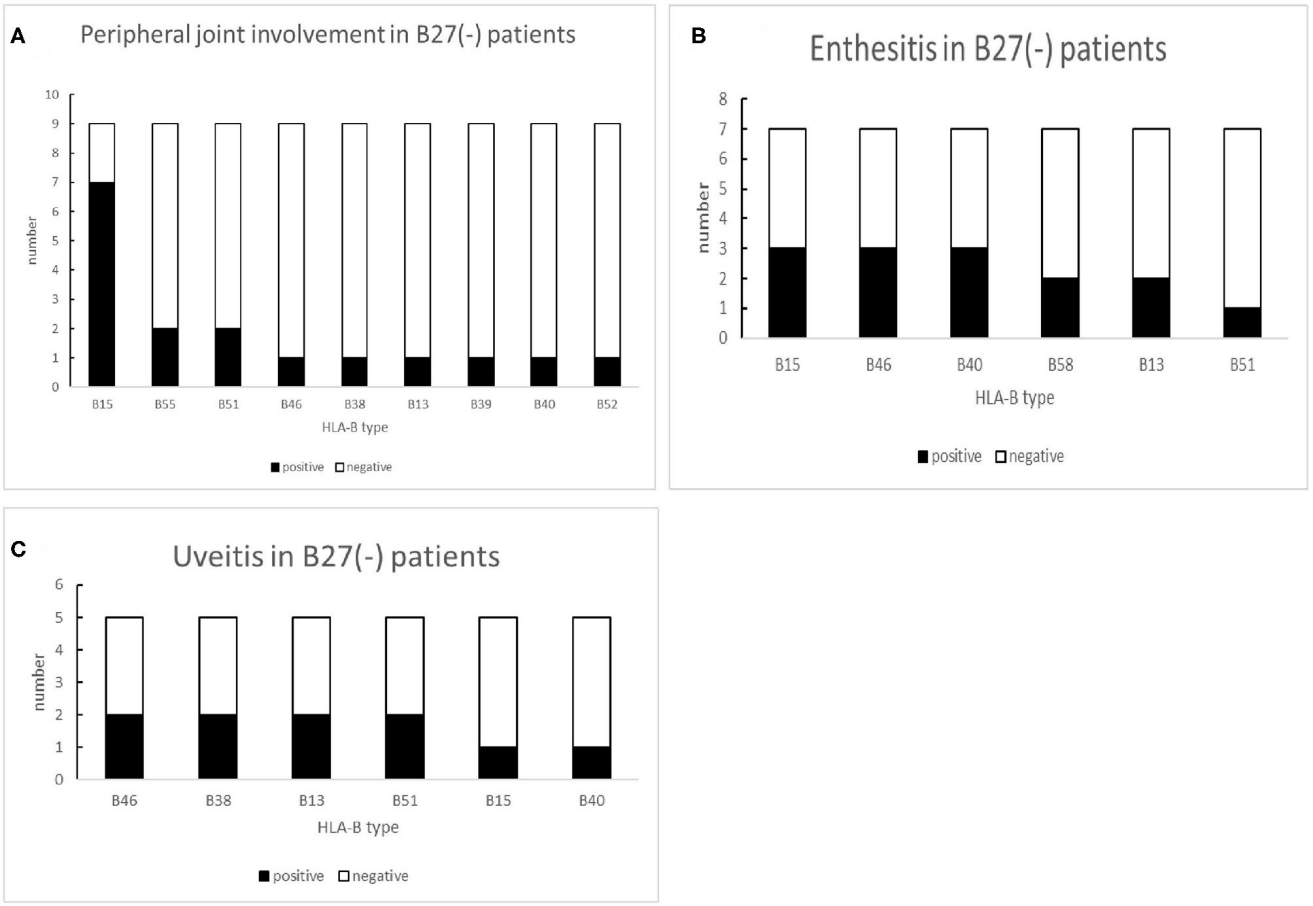
The number of other HLA-B types shown in B27(−) patients with different peripheral manifestations: **(A)** number of other HLA-B types shown in B27(−) patients with peripheral joint involvement, **(B)** number of other HLA-B types shown in B27(−) patients with uveitis, and **(C)** number of other HLA-B types shown in B27(−) patients with enthesis.

In nine B27(−) patients with peripheral joint involvement patients, seven cases (77.78%) carried HLA-B15. There was a significant difference in HLA-B15 (*p* = 0.014) (Table 6). For a small number of HLA-B27(−) patients with other manifestations (uveitis and enthesitis), the HLA-B type distributions are shown in Figure 6. One patient with dactylitis has a HLA-B46/B58 in his HLA-B allele.

**Table 6:** HLA-B15 and HLA-B^*^15:02 had association with peripheral joint involvement in HLA-B27(−) AS patients (step 2).

	**Peripheral joint involvement**	**Without peripheral joint involvement**	***p***	**OR (95% CI)**
B15^(+)^ (*N =* 14)	7	7		
B15^(−)^ (*N =* 22)	2	20	0.014 (Fisher)	10 (1.67–60.00)
B*15:02^(+)^ (*N =* 9)	5	4		
B*15:02^(−)^ (*N =* 27)	1	26	0.002 (Fisher)	32.5 (2.974–355.116)

#### The Clinical Manifestations in HLA-B27 Subtypes Homozygote and Heterozygote AS Patients

The most frequent subtype was HLA-B^*^27:04 in the B27(+) group, so we further analyzed the clinical manifestations of HLA-B^*^27:04 homozygous and heterozygous AS patients. One of the HLA-B^*^27:04/B^*^27:04 patients was a woman who only had the axial phenotype. An X-ray showed that the front edge of each vertebra of the spine was straight, the facet joints of the thoracolumbar and lumbar vertebra were blurred, that there was bony ankylosing, scoliosis, atlantoaxial subluxation, bilateral sacroiliac joint fusion, narrowed hip space, osteoid destruction, and that the surface of the left ischial tuberosity was rough.

In HLA-B^*^27:04 heterozygous AS patients, HLA-B^*^27:04/B^*^40:01 was the most frequent genotype (*N* = 35), and the second was HLA-B^*^27:04/B^*^46:01(*N* = 24). But HLA-B^*^27:04/B^*^46:01 was significantly more frequent in patients with peripheral joint involvement compared to HLA-B^*^27:04/B^*^40:01 (*p* = 0.007, OR=4.833, 95% CI 1.472–15.867). The same to patients with enthesis (*p* = 0.037, OR=3.452, 95% CI 1.044–11.420) (Table 5). We did not find other significant peripheral manifestations. One patient with HLA-B^*^27:04/B^*^35:05 had all peripheral manifestations (uveitis, peripheral joint involvement, dactylitis, and enthesitis).

All HLA-B^*^27:05 samples were heterozygote patients. Seventeen patients had clinical information. Among five cases (29.4%) with arthritis, two patients were HLA-B^*^27:05/B^*^46:01, and one patient was HLA-B^*^27:05/B^*^40:01. There were no different clinical manifestations between B^*^27:04 and B^*^27:05 (Table 4). Other HLA-B27 subtypes were also heterozygous, and their clinical peripheral phenotypes are shown in Table 3. We found one HLA-B^*^27:02/B^*^15:01 patient with all the peripheral manifestations.

#### The Clinical Manifestations in HLA-B27(−) Homozygote and Heterozygote AS Patients

According to high-resolution HLA-B genotypes, in the 36 B27(−) cases with clinical information, the highest HLA-B type was HLA-B^*^46:01 (*N* = 11, 30.56%). HLA-B^*^15:02, HLA-B^*^13:01, HLA-B^*^38:02, and HLA-B^*^40:01 were tied for second (*N* = 6). As mentioned earlier, patients with peripheral joint involvement carried HLA-B15 more frequently, especially HLA-B^*^15:02, which also has an association with the phenotype in B27(−) patients [*p* = 0.002_Fisher_, OR=32.5, 95% CI (2.974–355.116)] (Table 6). However, the patient with HLA-B^*^15:02 homozygote was a man who only had axial manifestations in the same way as the HLA-B^*^27:04 homozygote patient.

## Discussion

HLA-B27 as the major gene was closely related to the development of AS. The most prevalent subtypes are HLA-B^*^27:04 and HLA-B^*^27:05 in different populations. Other subtypes associated with the disease are B27:02, B^*^27:15, and so on. In our data, there were B^*^27:04, B^*^27:05, B^*^27:02, and B^*^27:15 in the patient group, and 88.9% of the patients were HLA-B^*^27:04. HLA-B27 positive patients had an earlier disease onset and higher family aggregation (12). HLA-B27 negative patients had a higher frequency of extra-spine manifestations (12). Research about Korean AS patients found that HLA-B27 homozygosity has no significant difference with heterozygosity on the clinical manifestations and radiographic progression (7, 8). Some research found only four homozygous of B^*^27:04 in 245 HLA-B27-positive AS patients (13). In our study, we found two HLA-B^*^27:04 homozygous, one HLA-B^*^27:04/B^*^27:14, and no homozygote of HLA-B^*^27:05, 27:02, or 27:15. Only two patients had axial symptoms. Perhaps other factors were associated with peripheral manifestations in AS patients.

In HLA-B27(+) patients, 20.2% of alleles showed as HLA-B40, and the primary subtype was HLA-B^*^40:01. And 16.9% of HLA-B^*^27:04 cases were HLA-B^*^27:04/B^*^40:01, which were not found in B27(+) controls. Samples with HLA-B^*^40:01 in HLA-B27(−) controls were more than B27(−) cases—perhaps as a result of the sample size. Other research found that 18.2% of AS patients carried B27/B40 and only 0.4% in healthy controls (14). HLA-B40 can increase HLA-B27 susceptibility to AS (15, 16). The different subtypes had different peripheral manifestations.

HLA-B46 can increase the risk of severe sacroiliitis development related to Japanese psoriatic arthritis (PsA) patients (17). The HLA-A2-B46-DR8 haplotype has a relationship with the levels of complement components (18). HLA-B^*^46:01 was the only subtype of HLA-B46 found in our data. In HLA-B27 AS, the frequency was second to that of HLA-B40. Relative to other HLA-B alleles, patients with HLA-B^*^27:04/B^*^46:01 had a higher prevalence of peripheral joint involvement. HLA-B^*^46:01 was associated with peripheral joint involvement in HLA-B^*^27:04 AS patients.

In our data, 11.5% of HLA-B27 patients combined with HLA-B15 and 33.33% in HLA-B27(−) patients. The major subtype was HLA-B^*^15:02. HLA-B^*^15:17 was found in seven controls, not AS patients. In undifferentiated SpA, HLA-B15 was increased (19). HLA-B15 can be an independent factor of peripheral SpA (20). In HLA-B27 negative patients, HLA-B15, especially HLA-B^*^15:02, had a relationship with peripheral joint involvement in patients. HLA-B15 may increase the risk of peripheral joint involvement in HLA-B27 negative patients.

HLA-B35 was associated with AS (21, 22). Previous research found that seven HLA-B27(−) AS families with idiopathic inflammatory bowel disease have a higher frequency of HLA-B15 (21). All five HLA-B^*^27:04/B^*^35:01 were patients. Three HLA-B27(−) patients carried HLA-B^*^35:03. One heterozygous patient with B^*^35:05/B^*^27:04 had multiple peripheral symptoms of uveitis, enthesitis, peripheral joint involvement, and dactylitis.

Allele HLA-B51 is associated with Behcet's disease (23), especially in ocular involvement (24). But some results showed that HLA-B27(+)B51(+) is a good factor of Behcet uveitis (25). HLA-B51 was present in autoimmune diseases other than Behcet's disease with high prevalence (26). Eighteen cases showed HLA-B27/B51 (including B^*^51:01 and B^*^51:02). Only one patient with HLA-B27:04/B^*^51:01 had uveitis and dactylitis.

HLA-B38 was associated with clozapine-induced agranulocytosis (27). In the Argentine and Israeli population, the HLA-B38 was associated with PsA (28, 29). But psoriatic arthritis patients with HLA-B38 had less back pain (30). In our data, no patients with HLA-B27/B38 showed psoriatic arthritis.

Seventeen B27(+) patients showed B58. There was no difference between patients and controls. As we all know, Allopurinol-induced severe cutaneous adverse drug reactions (SCAR) is strongly associated with the presence of HLA-B^*^58:01 (31). But no article has yet reported the relationship between B^*^58:01 and AS. Further study is necessary to explore the association.

In the present study, we evaluated the HLA-B genotype in AS patients compared to the control group. As a result, we found that more than 98% of the samples were heterozygous in the HLA-B region. HLA-B27 homozygous patients were rare and only had axial manifestations. Based on our study and other reports, for B27(+) people, HLA-B40 can increase the risk of AS. HLA-B40 was the second most common HLA-B subtype in all of the AS patients besides HLA-B27. Then the genotype HLA-B27:04/B^*^40:01 can improve diagnostic accuracy, and patients with HLA-B^*^27:04/B^*^46:01 had a high risk of arthritis and enthesitis. In the HLA-B27(−) group, HLA-B^*^15:02 was a risk maker of peripheral joint involvement. Perhaps HLA-B^*^40:01, HLA-B^*^46:01, and HLA-B^*^15:02 should be markers included in AS diagnosis value. Due to the limited information in this field and a small number of patients, our results did not show statistical significance in other HLA-B subtypes with peripheral clinical manifestations. There is a need for more samples and further workup on the relationship of the HLA-B heterozygous in AS patients.

In conclusion, our research shows that, besides HLA-B27, other HLA-B types also may impact the AS patient phenotype. It is critical to systematically screen and evaluate the HLA-B genotype in the patients with AS, which may result in an improved accurate diagnosis of the patients.

## Data Availability Statement

The authors acknowledge that the data presented in this study must be deposited and made publicly available in an acceptable repository, prior to publication. Frontiers cannot accept a manuscript that does not adhere to our open data policies.

## Ethics Statement

The studies involving human participants were reviewed and approved by The ethics committee of Third Affiliated Hospital of Sun Yat-Sen University. The patients/participants provided their written informed consent to participate in this study.

## Author Contributions

JG conceived the study and critically revised the manuscript and provided final approval of the manuscript. JW, PZ, and XL were in charge of the experiment. XW performed the analysis. XZ, ZC, QL, LT, QW, and SC were in charge of collecting sample and data. XW wrote the first draft of the manuscript. All authors contributed to the article and approved the submitted version.

## Conflict of Interest

The authors declare that the research was conducted in the absence of any commercial or financial relationships that could be construed as a potential conflict of interest.
