# Supplementary material for: The Clinical Characteristics of Other HLA-B Types in Chinese Ankylosing Spondylitis Patients
Source: Front Med (Lausanne). 2021 Jan 8;7:568790. doi: 10.3389/fmed.2020.568790 (PMC7820707; doi:10.3389/fmed.2020.568790)
Supplement: Supplementary file 1 [file Table_1.DOCX]

| Table S1 the Number of other HLA-B genotypes in HLA-B27 positive samples | | | | | | | |
| --- | --- | --- | --- | --- | --- | --- | --- |
|  | B27(+) AS | | | | | | B27(+) HC |
|  | B*27:02 | B*27:04 | B*27:05 | B*27:06 | B*27:07 | B*27:15 | B*27:04 |
| B*07:02 |  | 2 | 1 |  |  |  |  |
| B*07:05 |  | 1 |  |  |  |  |  |
| B*08:01 |  | 2 |  |  |  |  |  |
| B*13:01 |  | 16 | 1 | 1 |  |  | 2 |
| B*13:02 |  | 5 | 2 |  |  |  |  |
| B*15:01 | 2 | 4 |  |  |  |  | 1 |
| B*15:02 |  | 15 | 1 |  |  |  | 1 |
| B*15:11 |  | 1 | 1 |  |  |  |  |
| B*15:12 |  | 3 | 1 |  |  |  |  |
| B*15:25 |  | 1 |  |  |  |  |  |
| B*18:02 |  |  |  |  |  |  | 1 |
| B*27:04 |  | 2 |  |  |  |  |  |
| B*27:14 |  | 1 |  |  |  |  |  |
| B*35:01 |  | 5 |  |  |  |  |  |
| B*35:03 |  | 1 |  |  |  |  |  |
| B*35:05 |  | 2 |  |  |  |  |  |
| B*37:01 |  | 2 |  |  |  |  | 1 |
| B*38:01 |  | 3 |  |  |  |  |  |
| B*38:02 |  | 9 |  |  |  |  | 4 |
| B*39:01 |  | 4 |  |  |  | 1 | 2 |
| B*39:05 |  |  |  |  |  |  | 1 |
| B*40:01 |  | 38 | 3 |  |  |  |  |
| B*40:02 |  | 5 |  |  |  |  | 1 |
| B*40:06 |  | 5 |  |  |  |  |  |
| B*41:01 |  | 1 |  |  |  |  |  |
| B*44:02 |  |  |  |  |  |  | 1 |
| B*44:03 |  |  | 1 |  |  |  |  |
| B*46:01 | 1 | 27 | 6 |  |  | 1 | 6 |
| B*48:01 |  | 6 |  |  |  |  | 2 |
| B*48:03 |  |  | 1 |  |  |  |  |
| B*51:01 |  | 12 | 1 |  |  |  | 2 |
| B*51:02 |  | 5 |  |  |  |  |  |
| B*52:01 |  | 4 | 1 |  |  |  |  |
| B*52:25 |  | 1 |  |  |  |  |  |
| B*54:01 |  | 8 |  |  |  |  | 2 |
| B*55:02 |  | 9 |  |  |  |  | 1 |
| B*55:12 |  | 1 |  |  |  |  |  |
| B*56:01 |  | 6 |  |  |  |  |  |
| B*57:01 |  |  | 1 |  |  |  |  |
| B*58:01 |  | 16 |  |  | 1 |  | 3 |
| B*67:01 |  | 1 |  |  |  |  | 1 |
| total number | 3 | 224 | 21 | 1 | 1 | 2 | 32 |

AS ankylosing spondylitis; HC health controls

| Table S2the Number of other HLA-B genotypes in HLA-B27 negative samples | | |
| --- | --- | --- |
|  | HLA-B27(-) AS | HLA-B27(-) HC |
| B*07:02/B*40:01 | 0 | 1 |
| B*07:02/B*46:01 | 2 | 1 |
| B*07:02/B*52:01 | 1 | 0 |
| B*07:05/B*13:01 | 1 | 0 |
| B*07:05/B*15:02 | 1 | 0 |
| B*07:05/B*46:01 | 0 | 1 |
| B*08:01/B*52:01 | 0 | 1 |
| B*13:01/B*15:02 | 1 | 1 |
| B*13:01/B*15:08 | 1 | 0 |
| B*13:01/B*38:02 | 0 | 2 |
| B*13:01/B*39:01 | 0 | 1 |
| B*13:01/B*40:01 | 1 | 3 |
| B*13:01/B*40:06 | 0 | 2 |
| B*13:01/B*46:01 | 1 | 1 |
| B*13:01/B*51:01 | 1 | 1 |
| B*13:01/B*55:02 | 1 | 1 |
| B*13:02/B*44:02 | 1 | 0 |
| B*13:02/B*51:01 | 2 | 0 |
| B*15:01/B*46:01 | 2 | 0 |
| B*15:01/B*49:01 | 0 | 1 |
| B*15:01/B*55:02 | 1 | 0 |
| B*15:01/B*58:01 | 0 | 1 |
| B*15:02/B*15:02 | 1 | 0 |
| B*15:02/B*15:11 | 1 | 0 |
| B*15:02/B*15:25 | 1 | 0 |
| B*15:02/B*38:02 | 1 | 0 |
| B*15:02/B*39:01 | 1 | 0 |
| B*15:02/B*40:01 | 1 | 0 |
| B*15:02/B*40:02 | 0 | 1 |
| B*15:02/B*46:01 | 1 | 1 |
| B*15:02/B*51:01 | 1 | 0 |
| B*15:02/B*52:01 | 0 | 1 |
| B*15:02/B*55:02 | 1 | 0 |
| B*15:07/B*35:67 | 0 | 1 |
| B*15:07/B*40:02 | 0 | 1 |
| B*15:11/B*54:01 | 0 | 1 |
| B*15:12/B*38:02 | 1 | 0 |
| B*15:12/B*40:01 | 0 | 1 |
| B*15:12/B*52:01 | 1 | 0 |
| B*15:17/B*15:18 | 0 | 1 |
| B*15:17/B*46:01 | 0 | 2 |
| B*15:17/B*48:01 | 0 | 2 |
| B*15:17/B*57:01 | 0 | 1 |
| B*15:17/B*67:01 | 0 | 1 |
| B*15:18/B*15:27 | 1 | 0 |
| B*15:18/B*46:01 | 1 | 0 |
| B*15:25/B*40:01 | 2 | 1 |
| B*15:27/B*35:01 | 1 | 0 |
| B*35:01/B*40:01 | 0 | 1 |
| B*35:01/B*40:06 | 1 | 0 |
| B*35:03/B*38:02 | 1 | 0 |
| B*37:01/B*40:01 | 1 | 0 |
| B*38:02/B*40:01 | 1 | 1 |
| B*38:02/B*40:06 | 1 | 0 |
| B*38:02/B*46:01 | 3 | 0 |
| B*38:02/B*51:02 | 1 | 0 |
| B*38:02/B*54:01 | 1 | 1 |
| B*38:02/B*55:02 | 1 | 0 |
| B*39:01/B*40:01 | 0 | 1 |
| B*39:01/B*46:01 | 1 | 0 |
| B*39:01/B*67:01 | 1 | 0 |
| B*40:01/B*40:01 | 0 | 2 |
| B*40:01/B*46:01 | 0 | 3 |
| B*40:01/B*48:01 | 0 | 1 |
| B*40:01/B*48:03 | 0 | 1 |
| B*40:01/B*51:01 | 1 | 2 |
| B*40:01/B*54:01 | 1 | 1 |
| B*40:01/B*56:02 | 1 | 0 |
| B*40:02/B*49:01 | 0 | 1 |
| B*40:02/B*55:02 | 1 | 0 |
| B*44:03/B*46:01 | 1 | 0 |
| B*46:01/B*46:01 | 1 | 0 |
| B*46:01/B*48:01 | 1 | 0 |
| B*46:01/B*51:01 | 2 | 1 |
| B*46:01/B*54:01 | 1 | 1 |
| B*46:01/B*55:02 | 2 | 1 |
| B*46:01/B*56:01 | 0 | 1 |
| B*46:01/B*58:01 | 2 | 2 |
| B*51:01/B*54:01 | 1 | 0 |
| B*51:01/B*55:02 | 1 | 0 |
| B*51:02/B*52:01 | 0 | 1 |
| B*54:01/B*58:01 | 0 | 1 |
| B*55:01/B*58:01 | 1 | 0 |
| B*55:02/B*67:01 | 1 | 0 |
| B*56:01/B*58:01 | 0 | 1 |
| B*58:01/B*58:01 | 2 | 0 |
| Total number | 66 | 57 |
